# A compressed sensing neuromorphic processor for sparse signal classification

**DOI:** 10.3389/fnins.2026.1777090

**Published:** 2026-05-21

**Authors:** Liyu Qian, Zikai Zhu, Yuhan He, Jie Lu, Yaojie Sun, Jiahui Guo, Lirong Zheng, Zhuo Zou

**Affiliations:** 1 State Key Laboratory of Integrated Chips and Systems, School of Information Science and Technology, Fudan University, Shanghai, China; 2 Department of Psychology, School of Social Development and Public Policy, Fudan University, Shanghai, China

**Keywords:** compressed sensing (CS), end-to-end, field programmable gate arrays (FPGA), neuromorphic processor, spiking neural network (SNN)

## Abstract

This paper presents a neuromorphic processing system integrating a compressed sensing spiking neural network (CSSNN) designed for sparse signal classification. The proposed CSSNN combines data coding, data compression, and SNN classification, enabling end-to-end optimization of network performance and model compression. Evaluated on the MNIST, N-MNIST, and DVS Gesture datasets, under uniform compression ratios (CRs) of 0.1, 0.05, 0.025, and 0.01, the proposed CSSNN consistently reduces the total number of network operations (OPs) by at least 80% compared with compressed learning methods using fixed Gaussian random matrix (GRM) sampling matrices, while maintaining minimal accuracy loss. A specialized CSSNN processor is designed based on a spike-driven processing flow. Validated on field-programmable gate arrays (FPGAs) and evaluated in the 40 nm CMOS process for application-specific integrated circuit (ASIC) design, this CSSNN processor achieves 96.12% classification accuracy with 8-bit fixed-point quantization on the MNIST dataset. The energy consumption of the ASIC is estimated to be 2.089 mW under a 1.1-V supply voltage and 100 MHz frequency.

## 1 Introduction

There is a growing demand for deploying artificial intelligence (AI) on edge devices. When deployed on edge platforms, spiking neural networks (SNNs) demonstrate superior energy efficiency and resource utilization compared with conventional artificial neural networks (ANNs), including deep neural networks (DNNs). The efficiency of SNNs originates from the spike-based operational mechanism of the cerebral cortex, which processes visual information through spatiotemporally sparse neuronal spikes (Maass, 1997).

Inspired by the success of traditional ANNs, several deep SNN models have been proposed (Yang et al., 2020; Srinivasan and Roy, 2019; Kuang et al., 2021). These large-scale models have achieved remarkable accuracy across various image classification tasks. However, this performance is attained at the cost of thousands to millions of neurons and millions to billions of synapses, resulting in substantial computational and memory overhead.

To address the constraints of resource-limited edge devices, lightweight SNN architectures implemented on small-scale neuromorphic application-specific integrated circuit (ASIC) systems with limited spiking neurons and synapses have been developed (Frenkel et al., 2018; Kim et al., 2020; Besrour et al., 2023; Frenkel and Indiveri, 2022). In this context, field-programmable gate arrays (FPGAs) are widely adopted as flexible platforms for rapid validation of SNN models and neuromorphic hardware architectures.

Lightweight SNN models commonly adopt generic compression techniques originally developed for DNNs, such as weight pruning (Chen et al., 2023) and quantization methods (Li et al., 2025). To compensate for the accuracy loss introduced by model compression, some approaches, such as online learning mechanisms, are introduced, which require additional computational and hardware overhead.

The biological nature of SNNs inherently incorporates sparsity observed in biological neural systems, characterized by sparse coding (Foldiak, 2003; Spanne and Jörntell, 2015), sparse connectivity (Faghihi et al., 2022), and event-driven spiking and computation (Brette, 2012). Sparse coding aims to represent input data as a sparse linear combination of basis functions (receptive fields), enabling the extraction of high-level and informative features. Sparse coding has been widely applied in compressed sensing (CS) and signal recovery tasks (Candes and Tao, 2006). When explicitly leveraged in dedicated hardware implementations, these properties lead to substantial computational savings.

A typical lightweight SNN model consists of an encoder layer, an input layer, one or more hidden layers, and an output layer. Common encoding methods in SNNs include temporal coding (Paugam-Moisy and Bohte, 2012), rate coding (Meftah et al., 2013), and others. Temporal coding converts data into a single spike with a precise timing delay (Engel et al., 1992). Although this coding method achieves high sparsity, it is sensitive to noise. Rate coding represents the data through the firing rates of neurons, but this robustness comes at the expense of higher firing rates and reduced sparsity (Guo et al., 2021). For image processing tasks, some studies integrate data encoding with feature extraction. In Yu et al. (2013), a supervised learning rule combined with temporal coding is proposed to generate spikes. In Liu and Yue (2017), spike-timing-based coding schemes, such as Rank Order Coding (ROC), are adopted to translate features into spiking patterns for fast visual information processing. However, these encoding methods are generally too complex to be implemented on-chip. Consequently, the encoder layer, which converts analog signals into neuronal spike trains, is often realized off-chip.

To realize an end-to-end lightweight SNN with high classification accuracy, we propose a compressed sensing–inspired spiking neural network (CSSNN) framework. The proposed system consists of a CS-inspired encoder and a three-layer SNN classifier, following a task-driven compressed learning paradigm. The encoder is integrated as the input layer to jointly perform data encoding, compression, and discriminative feature extraction.

The compression process is fundamentally sampling-oriented: by sampling the raw input using a structured, CS-inspired scheme, sub-Nyquist acquisition is achieved, enabling efficient data compression and reducing the overall model size while preserving task-relevant information.

To address the accuracy degradation introduced by model compression, we further propose a co-optimization algorithm that jointly updates the CS encoder and the SNN classifier. In addition, hardware-friendly optimizations are applied to the proposed model, including sparsity enhancement and quantization. In particular, following a compressed learning–inspired design, the CS encoder adopts a binary {0, 1} measurement matrix that models a structured sampling process, enabling efficient sparse connectivity and facilitating hardware implementation.

A specialized energy-efficient neuromorphic processor is designed based on the proposed CSSNN model, supporting fully on-chip data encoding and classification. The proposed model is fully implemented on a flexible FPGA platform, enabling efficient classification of raw image data. The contributions and novelty of this work are summarized as follows:

- We propose an end-to-end CSSNN framework that enables on-chip data encoding and model compression. Different from conventional CS-based pipelines, the CS encoder is not treated as a fixed or independent preprocessing module but is tightly integrated as the input layer of the network and jointly optimized with the SNN classifier. Inspired by compressed learning (CL), the framework performs task-adaptive co-design of sensing and classification, where the measurement process is directly aligned with the classification objective rather than signal reconstruction. This joint optimization is realized via spatio-temporal backpropagation (STBP), allowing the model to achieve high compression ratios with minimal accuracy degradation.
- We introduce a hardware-aware optimization strategy that jointly considers sparsity, computational efficiency, and physical realizability. Unlike conventional learned encoders that rely on unconstrained real-valued projections or BNN-style binarization for weight approximation, the CS encoder employs a binary {0, 1} measurement matrix that explicitly models the sampling mechanism in compressed sensing. In this formulation, a value of 1 denotes a physical sampling operation rather than a weight contribution. Furthermore, a column-wise constraint is imposed such that each measurement samples a fixed number of input elements, forming a structured sub-sampling operator with a fixed sensing budget. Based on this design, we develop a sparsity adjustment function that optimizes the measurement matrices through pruning and binarization without modifying the SNN architecture. This enables flexible trade-offs among classification accuracy, spike activity, and computational cost, while substantially reducing model size. Experimental results on MNIST, N-MNIST, and DVS Gesture show the total number of network operations (OPs) reductions of at least 80% compared with compressed learning method using fixed Gaussian random matrix (GRM) sampling matrix, while maintaining minimal accuracy loss.
- We design a dedicated CSSNN processor to support fully on-chip data classification. The processor integrates a CS encoder module and an SNN module, where the CS encoder implements the structured sparse binary measurement matrix using an index-matching scheme, directly realizing the sampling operation in hardware. Benefiting from the non-negative accumulation property of the {0, 1} measurement matrix, the design avoids sign handling and enables efficient hardware-friendly accumulation. The SNN module adopts a spike-driven processing flow to exploit both input and activation sparsity for low-power computation. The proposed processor achieves on-chip encoding without external pre-processing and is validated on FPGA followed by ASIC implementation in 40 nm CMOS technology. The chip achieves a power consumption of 2.089 mW at 1.1 V supply and 100 MHz operating frequency.

## 2 Methodology

In this section, we present the CSSNN system for sparse signal classification. The proposed system consists of two stages: a CS encoder module for data coding and compression, and feature extraction, followed by an SNN classifier module for data classification.

Two techniques are applied to realize a lightweight SNN for resource-constrained classification tasks while maintaining high classification accuracy. First, the measurement matrix of the CS encoder is implemented as a fully connected (FC) input layer and co-optimized with the SNN classifier using the STBP algorithm to improve classification performance. Second, a sparsity adjustment function is applied to the measurement matrix through a combination of pruning and binarization. This hardware-aware optimization enables adjustable computational complexity in the CS encoder layer and controllable neuron firing rates.

**Figure 1:**
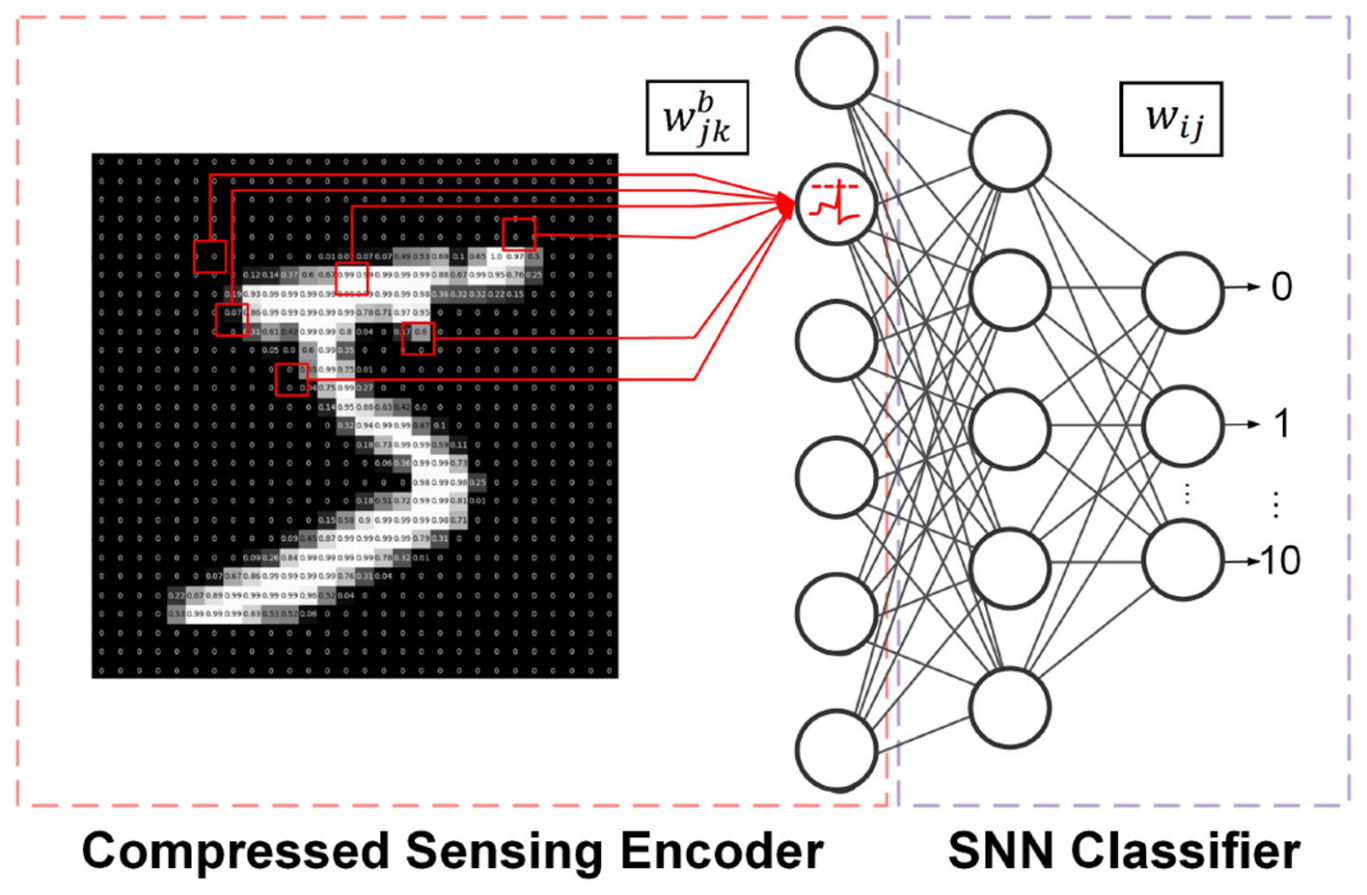
End-to-end process of the proposed CSSNN.

The end-to-end process of the proposed CSSNN is illustrated in Figure 1. The overall automatic network optimization for signal classification using the CSSNN method is outlined in Algorithm 1.

Algorithm 1 Automatic optimization procedure for CSSNN. 
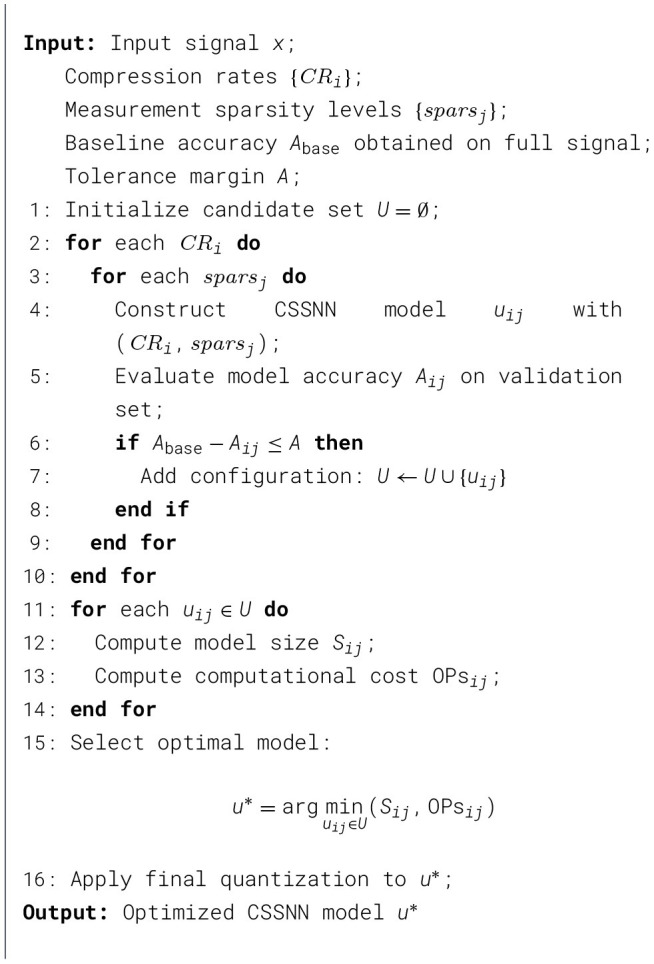

### 2.1 Fundamentals of compressed sensing and SNN

#### 2.1.1 Compressed learning algorithm

Compressed learning (CL) is a combination of CS and machine learning that implements classification directly on the low-dimensional measurement domain (Calderbank et al., 2009).

In CS, given the original signal *x*∈*R*^*n*^, the signal is compressed through a measurement matrix Φ∈*R*^*m*×*n*^ (such that *m*≪*n*) to obtain the compressed measurement vector $\hat{x}\in R^{m}$. The compression rate (CR) is $\frac{m}{n}$.

The sparse signal *x* can be recovered from $\hat{x}$. The compression process is demonstrated in Equation 1:

$$\begin{array}{l} \hat{x}=\Phi x+e \end{array}$$ (1)

In the CS theory, the measurement matrix Φ must be incoherent with respect to the sparse transformation basis Ψ so that the compressed signal can be reconstructed. Typically, a GRM or Bernoulli random matrix is the basic measurement matrix for all sparse signals.

The CL method saves the complex reconstruction process, processing directly on the compressed vector $\hat{x}$.

#### 2.1.2 Neuron model

Neurons are the basic working elements of the nervous system that propagate electrochemical signals through action potentials and pass on information (Yamazaki et al., 2022). A number of neuron models describing the timing of spikes and the variation of neuron membrane potential (MP) have been developed in the form of ordinary differential equations. The neuron models vary from the biologically accurate ones, such as the Hodgkin-Huxley model (Hodgkin and Huxley, 1952) to those more computationally feasible ones, such as the integrate-and-fire (IF) model (Izhikevich, 2004). Among these models, the leaky integrate-and-fire (LIF) model is widely adopted as a trade-off between biological accuracy and simplicity for computation.

The LIF model introduces the membrane leakage to the IF model as the reflection of the diffusion of ions through the membrane. The model dynamics of the neuron connected with *n* pre-synaptic neurons is demonstrated in Equation 2:


$$\begin{array}{l} v_{mem}^{\prime }=v_{mem}e^{-\frac{\Delta t}{\tau }}+\overset{N}{\underset{i=0}{\sum }}W_{i}u\left(t-t_{k}\right) \end{array}$$ (2)

where *v*_*mem*_ is the MP of the LIF neuron, $v_{mem}^{\prime }$ is the MP of the LIF neuron after the spike input from the pre-synaptic neurons and before the spike firing. τ is the time length of the membrane leakage. Δ*t* is the simulation time step. *W*_*i*_ is the weight matrix. *t*_*k*_ is the spike time of the pre-synaptic neurons. *u*(*t*) is the step function. When the MP reaches the threshold as *v*_*mem*_≥*v*_*th*_, the neuron fires the spike and resets as *v*_*mem*_ = *v*_*reset*_.

### 2.2 Proposed CSSNN system

### 2.2.1 CSSNN structure

As shown in Figure 1, the raw image data are directly fed into the CS encoder, where the signal dimensionality is reduced using a binarized measurement matrix. The compressed data are then accumulated as MPs by LIF neurons and converted into spikes that are forwarded to the SNN classifier. Compared with conventional lightweight FC SNNs, the proposed CSSNN significantly reduces the overall model size by employing a smaller input layer while enabling fully on-chip data encoding.

However, this compression inevitably introduces information loss and additional computational overhead associated with the CS process. To address these issues, an end-to-end co-optimization algorithm is introduced to enable classification-oriented signal sensing, together with a hardware-aware measurement matrix optimization strategy that effectively reduces the computational cost of the CS encoder.

### 2.2.2 The end-to-end co-optimization algorithm

According to Lotfi and Vidyasagar (2020), the binary matrix shows a great advantage in computation speed and storage requirements over typical measurement matrix such as GRM while achieving robust sparse recovery from the compressed signal.

Inspired by the binarized neural network (BNN) model, we realize the CS process in the form of an *m*×*n* FC layer with binarized elements.

The mathematical model of an artificial neuron of the BNN is shown as follows:


$$\begin{array}{l} y=f\left(\sum_{i}w_{i}x_{i}+b\right) \end{array}$$ (3)

where the neuron input *x*_*i*_ is the raw signal data, the corresponding weight *w*_*i*_ functions as the measurement matrix, the bias *b* functions as noise, the nonlinear activation function *f* functions as *f* = 1(*) and the output layer *y* is the compressed signal. All the elements of the measurement matrix *w*_*i*_ are binarized as $w_{i}^{b}\in \left\{0,1\right\}$.

The STBP algorithm is suggested as the training scheme to generate the SNN model. This algorithm models the continuous time *t* of the LIF dynamic process into multiple time units. The membrane leakage of the neuron is calculated as the multiplication of $v_{mem}e^{-\frac{1}{\tau }}$. The non-differentiable spike activity of the LIF neuron is approximated using surrogate functions. Propagating spikes layer-by-layer, the STBP algorithm extracts features of the SNN model from both spatial and temporal domains.

In the CSSNN method, the CS process is combined with an SNN classifier. We adopt the STBP algorithm to realize a co-optimization of the CS encoder and the SNN.

In the SNN layer, there is backpropagation (BP) in both the spatial and temporal domains. For each neuron in the SNN, the data flow of error propagation is similar to typical BP for DNNs. Each neuron accumulates the weighted error signals from the upper layer and iteratively updates the parameters in different layers. The CS encoder layer follows this process and updates the original weight *w* based on the layer-by-layer error calculated in the spatial domain.

**Figure 2:**
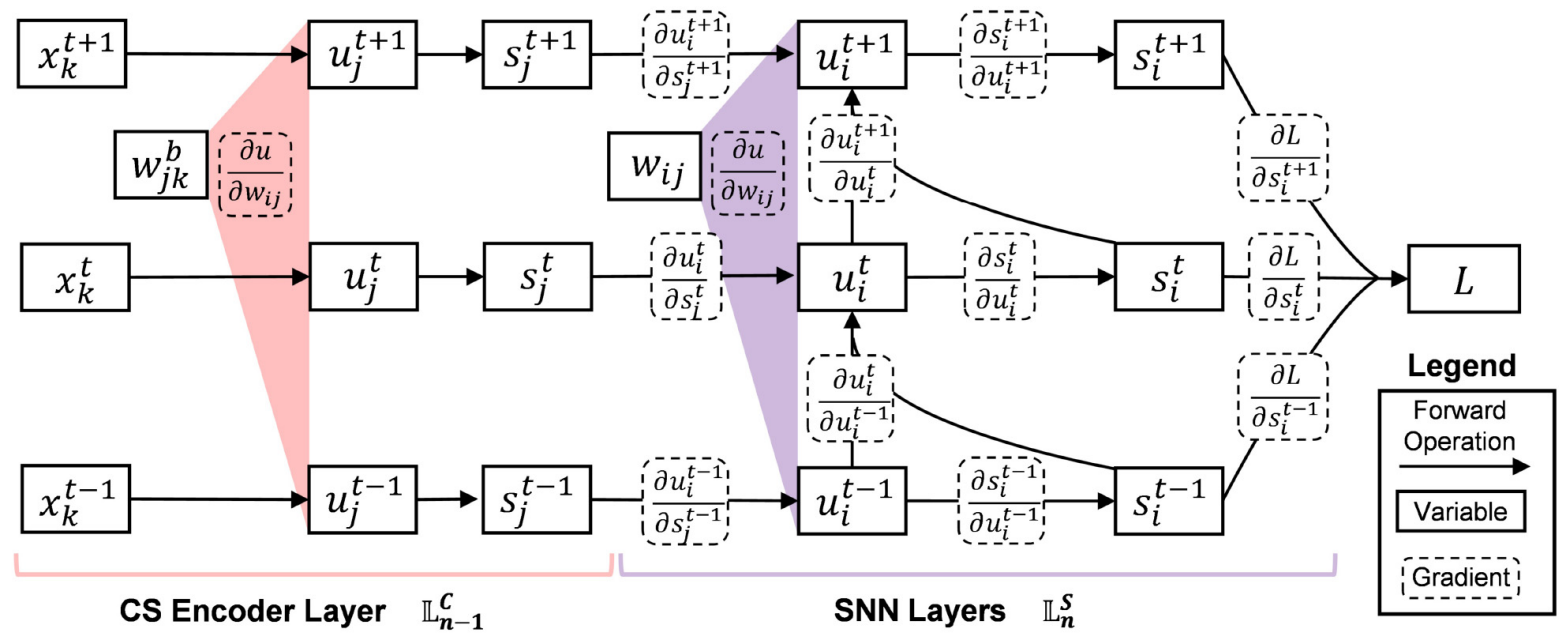
Detailed forward and backward process of the proposed CSSNN.

In the data flow of the time domain, a separate error is calculated to update the SNN weights only. The detailed forward and BP process of the CSSNN using the STBP algorithm is shown in Figure 2.

In Figure 2, solid lines denote the forward propagation process, while dashed lines represent the backward propagation (BP) based on surrogate gradients. Layer *k* corresponds to the input layer, layer *j* denotes the compressed sensing (CS) encoder layer, and layer *i* represents the spiking neural network (SNN) layer.

The input signal at layer *k* is denoted as $x_{k}^{t}$. In our setting, the input is assumed to be temporally shared across simulation steps within the inference window, i.e., $x_{k}^{t-1}=x_{k}^{t}=x_{k}^{t+1}$. The CS encoder produces the compressed representation $u_{j}^{t}$, which is implemented as a time-invariant spatial projection and thus remains constant across time steps. The corresponding spike output is denoted as $s_{j}^{t}$.

For the SNN layer, $u_{i}^{t}$ and $s_{i}^{t}$ represent the membrane potential (MP) and spike output at time step *t*, respectively. The SNN introduces temporal dynamics through membrane potential accumulation and spike generation.

During training, backpropagation in the spatial domain (SD) operates through both the CS encoder layer and the SNN layers, corresponding to the learnable sensing and classification pipeline. In contrast, backpropagation in the temporal domain (TD) is confined to the SNN layers, reflecting temporal credit assignment through the unfolded temporal dynamics of spiking neurons.

Algorithm 2 summarizes the training procedure of the proposed Binarized CSSNN. The network consists of a compressed sensing layer followed by SNN layers.

Algorithm 2 Binarized CSSNN training. 
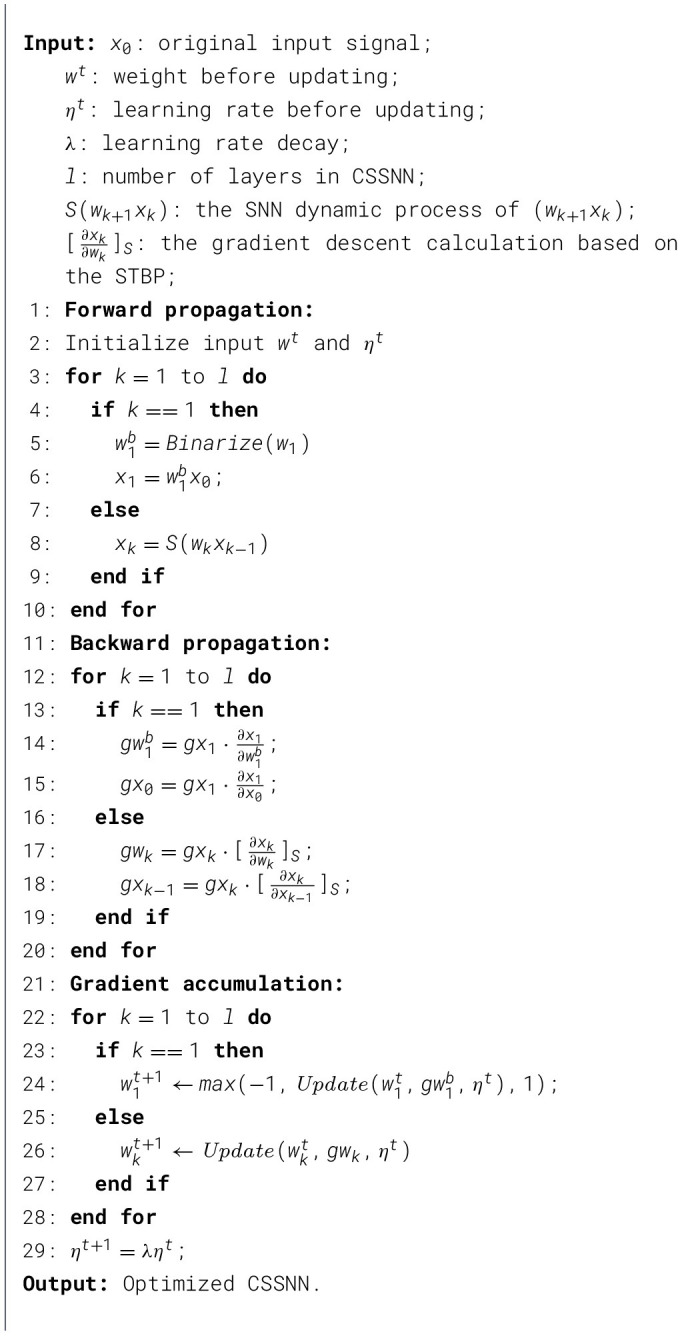

In the forward process, the first layer performs CS by first binarizing the learnable weight matrix *w*_1_ to obtain $w_{1}^{b}$. The binarization process is defined in Equation 4. The input signal *x*_0_ is then projected as $x_{1}=w_{1}^{b}x_{0}$, which serves as the compressed measurement. For subsequent layers (*k*>1), the feature propagation is governed by the SNN dynamics, i.e., *x*_*k*_ = *S*(*w*_*k*_*x*_*k*−1_), where *S*(·) denotes the spiking neuron model.

In the backward process, gradients are computed using STBP. For the CS encoder layer, gradients are propagated with respect to the original weight *w*_1_. For the SNN layers, gradients are approximated using surrogate functions denoted as [·]_*S*_ to handle the non-differentiability of spike activations.

During parameter update, the CS encoder layer weights are updated using gradient descent and then clipped to [−1, 1] to stabilize training. This clipping operation acts as an implicit regularization constraint and improves optimization stability. For all other layers, standard gradient updates are applied. The learning rate is further decayed by a factor λ at each iteration.

### 2.2.3 Hardware-aware measurement matrix optimization

As shown in Equation 3, the measurement matrix Φ of the CS process has a dimension of *m*×*n*, in which *n* stands for the length of the original signal and *m* stands for the length of the resized signal. All the elements of the matrix are binarized as $w_{i}^{b}\in \left\{0,1\right\}$. The number of ones determines the number of addition operations, thus affecting the power consumption greatly.

In this work, the number of ones in each row of the measurement matrix Φ∈*R*^*m*×*n*^ is marked as *Sample*. The sparsity of the measurement matrix is $spars=\frac{Sample}{n}$.

The function we use to binarize the weights is demonstrated in Equation 4:


$$w_{ij}^{b}=\{\begin{array}{ll} 1, & w_{ij}\in large(W_{j},Sample)\\ 0, & else \end{array}$$ (4)

where $w_{ij}^{b}$ is the binarized value of the original weight *w*_*ij*_. *W*_*j*_ is the *j*th row of the measurement matrix *W*. *large*(*W*_*j*_, *Sample*) is a set of *Sample* number of the largest elements from *W*_*j*_ and the small-weight connections are set to zero.

In the forward propagation process of CSSNN, the input signal is multiplied by $w_{ij}^{b}$, and the original weights *w*_*ij*_ are saved for updating. In the BP path, the loss function is computed between the output of the algorithm and the given target. In the gradient accumulation period, the CS encoder layer weights being updated are the original weights *w*_*ij*_.

The detailed binarized CS encoder layer training process is shown in Algorithm 2. This method enables dynamic performance-power consumption tuning in the model implementation. This model achieves different classification performance and power consumption without changing the SNN model structure.

For a CS encoder with *n* inputs and *m* outputs, the measurement matrix has a size of *m*×*n*. The original size of the binary matrix is calculated as *m*×*n*×*Datawidth*. The number of ones in the measurement matrix is *Sample*×*m*. In practice, the sparsity of the proposed measurement matrix is up to less than 1%.

The post-training quantization method is applied to the CSSNN model in order to reduce the storage overhead and enable hardware-friendly computation. The MPs of the neurons are quantized from 32-bit floating-point to 9-bit fixed-point, and the weights of the CSSNN are quantized to 8-bit fixed-point. The total model size is compressed by 4 × . The neuron MP storage is compressed by 3.55 × .

## 3 Proposed CSSNN processor design

### 3.1 Overall architecture

**Figure 3:**
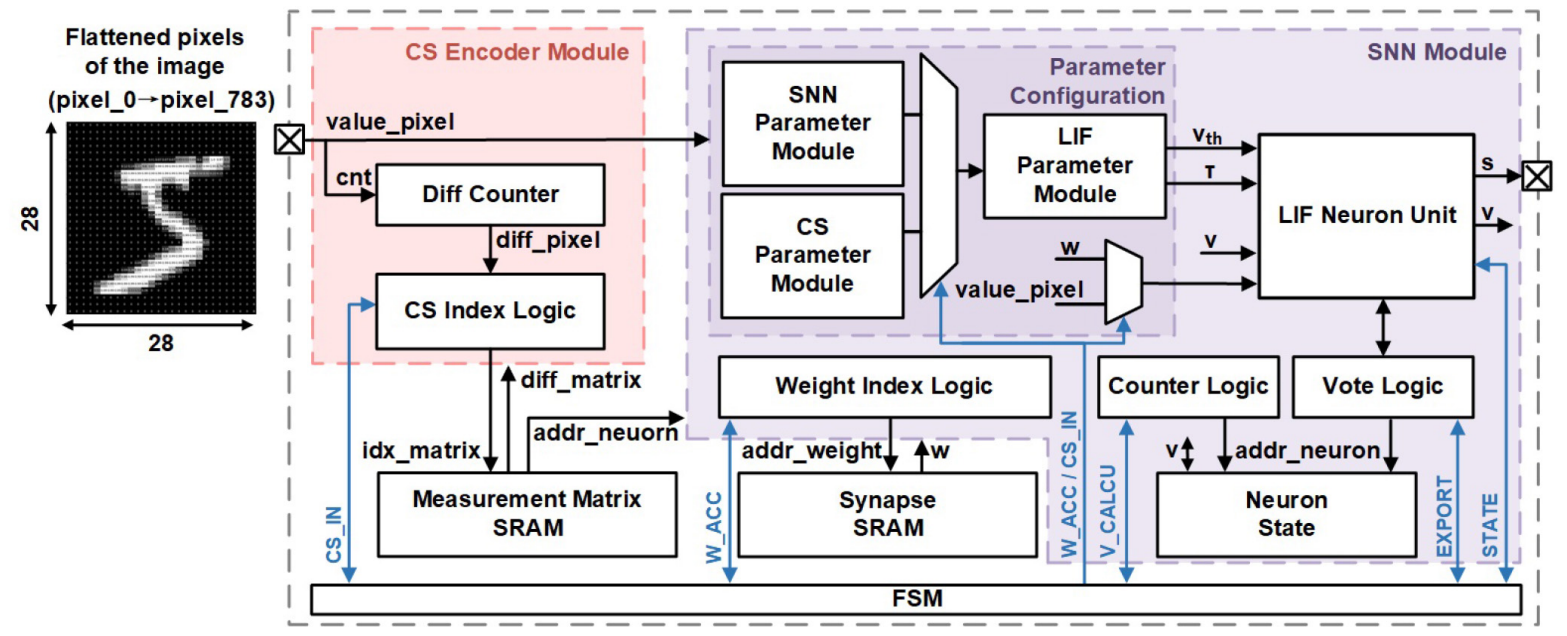
The proposed CSSNN processor architecture.

The overall architecture of the CSSNN processor is illustrated in Figure 3. The processor is composed of a CS encoder module, a SNN module, a measurement matrix SRAM, a synapse SRAM, a finite state machine (FSM), and a universal asynchronous receiver-transmitter (UART) interface.

The CS encoder module consists of an input pixel counter and CS index logic. Image pixel data are transmitted between the external system and the processor via the UART interface. The input pixel counter computes the index differences between the current input pixel and the previously sampled pixel, thereby generating a pixel address offset. The CS index logic retrieves the corresponding pixel index differences and neuron addresses from the measurement matrix SRAM, compares the index differences with the output of the pixel counter, and subsequently forwards the selected pixel value together with its associated neuron address to the SNN module.

The SNN module comprises a LIF neuron unit, a parameter configuration block, a weight index logic unit, a counter logic unit, a voting logic unit, and a neuron state storage unit. The LIF neuron unit performs MP computation under different operating modes configured by the finite state machine (FSM), and is responsible for spike generation and membrane potential updates. The parameter configuration block stores key neuron parameters, including the leakage factor τ and the firing threshold *V*_*th*_. The weight index logic unit manages synaptic weight access, while the counter logic unit controls the read and write operations of neuron membrane potentials. The voting logic unit aggregates and outputs spike events through the UART interface. The neuron state storage unit maintains the membrane potentials of all neurons. The overall inference workflow is orchestrated by the FSM. The synapse SRAM stores the synaptic weight of the SNN.

**Figure 4:**
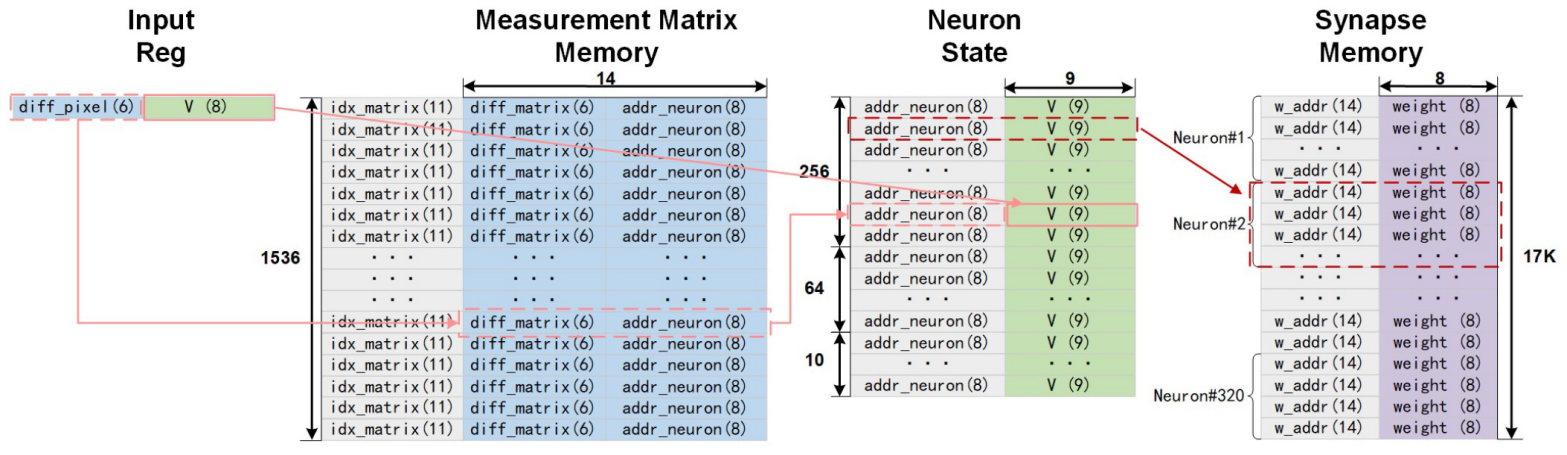
The memory organization and access scheme of the CS process and the SNN process following the spike-driven pre-neuron processing flow.

The memory organization and access scheme of the CS encoder and SNN modules in the spike-driven pre-neuron processing flow are illustrated in Figure 4. In this figure, the pink arrows denote the CS encoding flow, while the red arrows represent the SNN inference flow.

### 3.2 CS encoder module design

A CS encoder module is designed to perform the CS process for the serial input pixels in real time.

**Figure 5:**
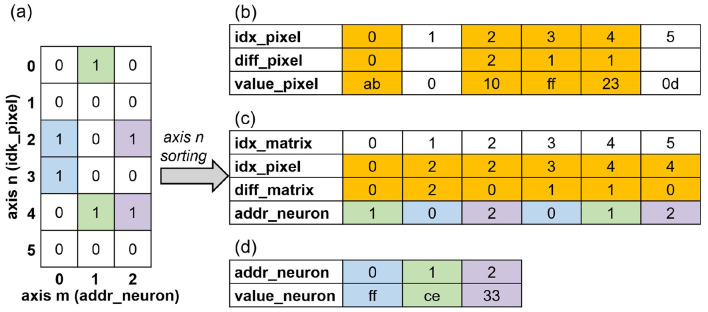
The index-matching flow of the CS encoder module. **(a)** Measurement matrix (binarized). **(b)** Input flattened pixels (8-bit fixed). **(c)** Relative index representation of the measurement matrix. **(d)** Compressed sensing pixels (8-bit fixed).

**Figure 6:**
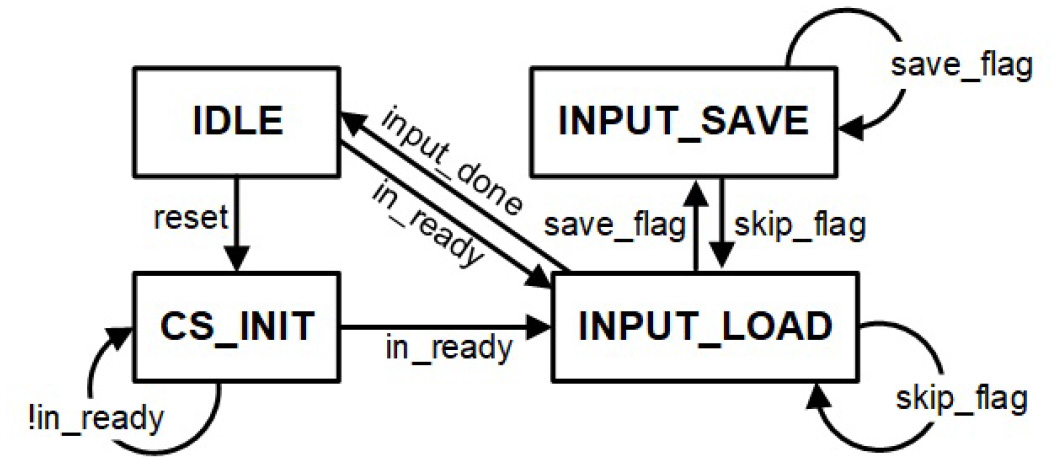
State transition diagram of the CS_FSM.

In this work, the index-matching method is employed to efficiently exploit the sparsity of the measurement matrix introduced by the hardware-aware measurement matrix optimization method demonstrated in Section 2. The processing procedure of the binarized measurement matrix with a dimension of *m*×*n* is demonstrated in Figure 5. The state transition diagram of the CS_FSM is shown in Figure 6.

Figure 5a shows a measurement matrix with a dimension of *m*×*n*. The nonzero entries in the same column are marked using identical colors. The matrix is first represented by the coordinates of the ones and then sorted along the axis *n*. The sorted matrix is shown in Figure 5c. Here, *idx*_*matrix* is the index of the sorted and flattened measurement matrix. *idx*_*pixel* is the index of the compressed sensing pixels along the axis *n*. *diff*_*pixel* is the relative difference of *idx*_*pixel*. *addr*_*neuron* is the index of the compressed sensing pixels along the axis *m*. The flattened input pixels are demonstrated in Figure 5b. The pixels marked in yellow correspond to the compressed sensing samples selected by the measurement matrix.

The CS_FSM in the CS encoder module consists of four states: *IDLE, CS*_*INIT, INPUT*_*LOAD*, and *INPUT*_*SAVE*. *IDLE* is the starting state. In the *CS*_*INIT* state, the diff counter block is initialized. Upon receiving image pixel data from the UART, the FSM transitions to the *INPUT*_*LOAD* state. In the *INPUT*_*LOAD* state, the UART sends in image pixels sequentially, and the diff counter block adds up *idx*_*pixel* by one. The CS index logic compares *diff*_*pixel* with *diff*_*matrix*, which is fetched from the measurement matrix SRAM according to *idx*_*matrix*. If the two values are equal, the CS_FSM switches to the *INPUT*_*SAVE* state. In the *INPUT*_*SAVE* state, the current input pixel value *value*_*pixel* and its corresponding neuron address *addr*_*neuron* are forwarded to the SNN module. The SNN module is configured to the *CS*_*IN* state. The MP of the addressed neuron stored in the neuron state block is updated accordingly. The resulting compressed sensing output is demonstrated in Figure 5d. The diff counter block resets *diff*_*pixel* to zero and adds up *idx*_*matrix* by one. The CS index logic block compares the updated *diff*_*pixel* and *diff*_*matrix*. If these two are equal, the CS_FSM remains in the *INPUT*_*SAVE* state; otherwise, the CS_FSM returns to the *INPUT*_*LOAD* state. After all entries in the measurement matrix SRAM have been processed, the CS operation is completed. The overall processing flow of the CS encoder module is demonstrated in Algorithm 3.

Algorithm 3 CS encoder module processing flow. 
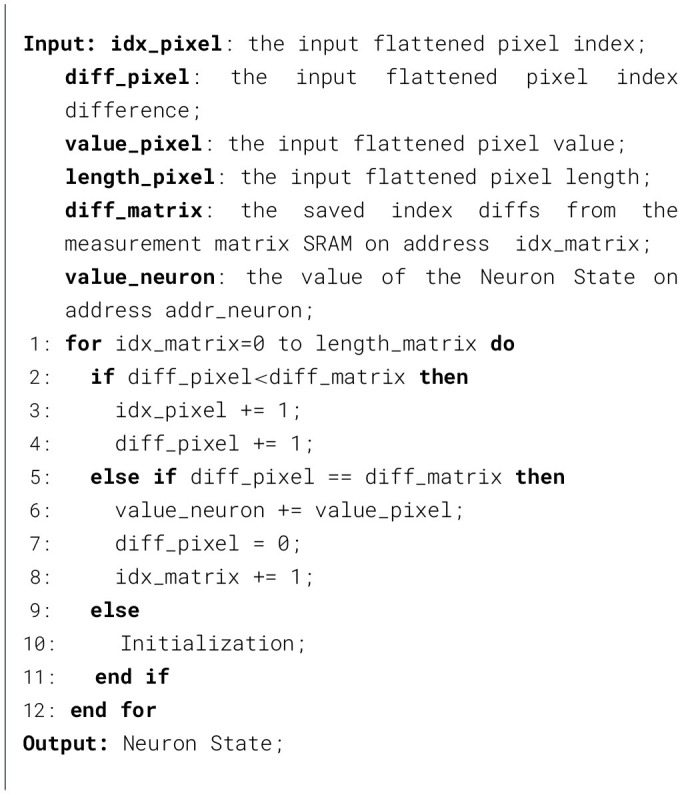

The resource utilization of the CS encoder module using the conventional FC matrix multiplication method and the index-matching method is compared in Table 1. In this evaluation, the parameters are set to *m* = 256, *n* = 784, *Sample* = 6, and *bits* = 14, corresponding to the CSSNN model described in Section 4.2. Compared with the conventional FC-based processing method, the proposed index-matching method reduces the parameter storage by a factor of 9.33 × . The number of weight read operations is reduced by 130.67 × , and the total computation cycles are reduced by 86.51 ×.

**Table 1:** CS encoder module resource utilization comparison.

Method	Binarized FC multiplication		Index-matching		Comparison
Weight storage	*m*×*n*×1	200,704	*m*×*Sample*×*bits*	21,504	9.33 ×
Weight read	*m*×*n*	200,704	*m*×*Sample*	1,536	130.67 ×
Cycles	*m*×*n*	200,704	*m*×*Sample*+*n*	2,320	86.51 ×

### 3.3 SNN module

**Figure 7:**
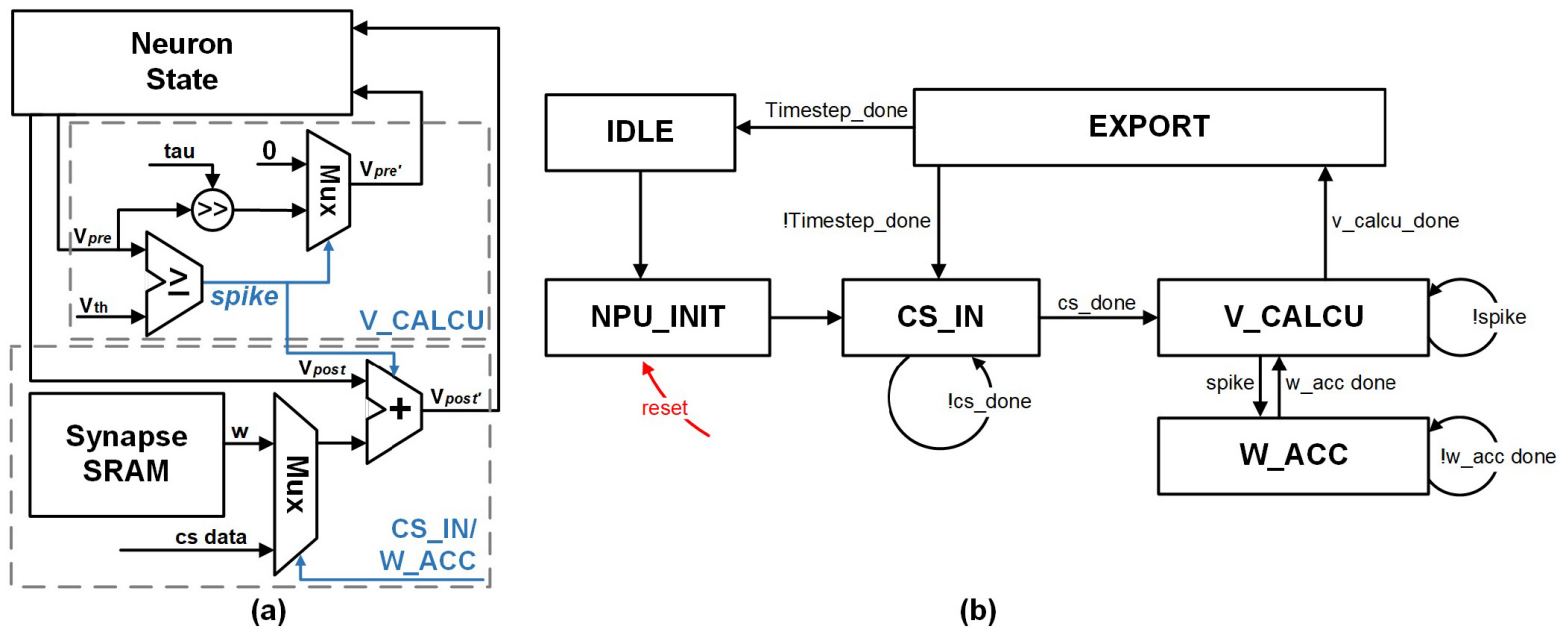
**(a)** The detailed structure of the LIF Neuron Unit. **(b)** The CSSNN FSM.

In the SNN module design, we employ the spike-driven pre-neuron processing flow, in which the synaptic weights are accumulated only when the pre-neurons fire spikes. The dynamics of the LIF neuron are demonstrated in Section 2.1. In the proposed SNN module, it is realized by the LIF neuron unit consisting of three parts: a multiplier for MP leakage, an adder for integrating weighted synaptic inputs to post-neurons, and a comparator for spike generation. The detailed structure of the LIF neuron unit is shown in Figure 7a. As marked in purple in Figure 3, the LIF neuron unit is configured to different states for spike firing and MP update by the CSSNN FSM demonstrated in Figure 7b.

The FSM in the SNN module consists of six states: *IDLE, NPU_INIT, CS_IN, V_CALCU, W_ACC*, and *EXPORT*. *IDLE* is the initial state. In the *NPU_INIT* state, all neuron states are initialized to zero. In the *CS_IN* state, the CS encoder module transmits the sampled CS data and the corresponding neuron address to the SNN module. The LIF neuron unit is configured to operate in the *CS_IN* state, where the adder inputs are the CS data and the MP taken from the neuron state block corresponding to the neuron address sent from the CS encoder module. The addition result is written back to the neuron state block. After all sampled image pixels are received, the FSM transitions to the neuron update state *V_CALCU*. In the *V_CALCU* state, the LIF neuron unit updates all neurons saved in the neuron state block sequentially. The comparator determines whether a neuron fires a spike. When a spike is generated, the neuron MP is reset to *v*_*reset*_, and the FSM transitions to the weight accumulation state *W_ACC*. The post-neurons are accumulated with weights fetched from the synapse SRAM via the weight index logic. When the neuron does not fire a spike, it is updated through the multiplier according to the leakage parameter τ. Once all the neurons have been processed, the FSM transitions to the *EXPORT* state to send the classification result through the UART. The FSM then returns to the *CS_IN* state and repeats this process for the predefined number of timesteps. The number of read and write operations for a CS encoder layer with a dimension of *m*×*n* are calculated as demonstrated in Equations 5 and 6.


$$\begin{array}{l} MeasurementMatrixRead=m\times Sample \end{array}$$ (5)

$$\begin{array}{l} NeuronRead/Write=m\times Sample \end{array}$$ (6)

The number of read and write operations of an SNN layer with a dimension of *m*×*n* are calculated as demonstrated in Equations 7 and 8.


$$\begin{array}{l} WeightRead=m\times n\times firingrate \end{array}$$ (7)

$$\begin{array}{l} NeuronRead/Write=m\times n\times firingrate+n \end{array}$$ (8)

Due to the low firing rate, the resource consumption is significantly reduced. The average firing rate of the proposed MNIST classification CSSNN is approximately 30%. The total number of read and write operations for the proposed MNIST classification task are calculated as 136,064 and 69,092, respectively.

## 4 Evaluation and analysis

In this section, the performance of the proposed CSSNN method is evaluated on three different datasets in terms of classification accuracy and computational complexity.

### 4.1 Datasets and evaluation standards

#### 4.1.1 Datasets

In this work, we evaluate our CSSNN method on three different datasets. The MNIST dataset contains handwritten digits in frame-based format (LeCun, 1998). The N-MNIST dataset is the spiking version of the original frame-based MNIST dataset. The N-MNIST dataset was captured by mounting the ATIS sensor on a motorized pan-tilt unit and having the sensor move while it views MNIST examples on an LCD monitor (Orchard et al., 2015). The IBM DVS Gesture dataset comprises 11 hand gesture categories from 29 subjects under three illumination conditions using a DVS camera (Amir et al., 2017). The detailed information of these datasets is shown in Table 2.

**Table 2:** Datasets details.

Name	Data form	Sensor	Class no.	Data size
MNIST	Frame image	-	10	28 × 28
N-MNIST	Spiking	ATIS	10	2 × 34 × 34
DVS gesture	Spiking	DVS camera	11	2 × 128 × 128

#### 4.1.2 Evaluation standards

In this work, we use the classification accuracy to evaluate the classification performance of the model, the model size to evaluate the hardware resource cost, and the number of operations (OPs) to evaluate the computational complexity. The definition of accuracy is shown below:


$$\begin{array}{l} \text{Accuracy}\;\left(\text{Acc}\right)=\frac{TP+TN}{TotalSamples} \end{array}$$ (9)

In Equation 9, Accuracy (Acc) denotes the overall classification accuracy, defined as the proportion of correctly classified samples among all evaluated samples. *TP* (true positives) represents the number of samples that are correctly predicted as belonging to the target class, while *TN* (true negatives) denotes the number of samples that are correctly predicted as not belonging to the target class. *TotalSamples* corresponds to the total number of samples in the evaluation set.

For multi-class classification, *TP* and *TN* are computed in a one-vs.-all manner, where each class is treated as the positive class and all remaining classes are collectively considered as the negative class.


$$\begin{array}{l} Modelsize=\frac{\sum_{i=1}^{l}\left(K_{H}\times K_{W}\times C_{in}\times C_{out}+b\right)\times bits}{8\times 1024\times 1024} \end{array}$$ (10)

In Equation 10, *Modelsize* denotes the total memory footprint of the model, measured in megabytes (MB). The summation is performed over all *l* layers of the CSSNN. For each layer, *K*_*H*_ and *K*_*W*_ represent the height and width of the convolutional kernel, respectively; *C*_*in*_ and *C*_*out*_ denote the numbers of input and output channels; and *b* is the number of bias parameters. The term *bits* indicates the bit width used for fixed-point representation (e.g., 8 bits). The numerator thus corresponds to the total number of stored parameters (weights and biases) multiplied by their bit precision, while the denominator converts the result from bits to megabytes. For fully connected layers, *K*_*H*_ = 1 and *K*_*W*_ = 1.

For the CS process, the measurement matrix is binarized with high sparsity and does not include bias terms. Accordingly, only the indices (addresses) of non-zero entries are stored for each row, leading to a compressed parameter representation.


$$\begin{array}{l} {Modelsize}_{\left[\text{CS}\right]}=\frac{\left(L_{in}\times CR\times Sample\right)\times bits}{8\times 1024\times 1024} \end{array}$$ (11)

In Equation 11, *Modelsize*_[CS]_ denotes the memory footprint of the CS encoder layer in MB. *L*_*in*_ is the length of the flattened input signal, and *CR* = *m*/*n* represents the compression rate, where *m* and *n* denote the numbers of measurements and original signal dimensions, respectively. *Sample* denotes the average number of non-zero entries retained per measurement (i.e., per row of the measurement matrix). The term *bits* corresponds to the bit width required to encode each index, which depends on *L*_*in*_ (i.e., ⌈log_2_*L*_*in*_⌉).

For MNIST, N-MNIST, and DVS Gesture datasets, the flattened input lengths are 784, 2,312, and 32,768, respectively, corresponding to index bit widths of 10, 11, and 15 bits.

The computational complexity in terms of operations (OPs) is defined as:


$$\begin{array}{l} OPs=\sum_{i=1}^{l}\left(2\times H_{out}\times W_{out}\times C_{out}\times C_{in}\times K_{H}\times K_{W}\right) \end{array}$$ (12)

In Equation 12, *OPs* denotes the total number of arithmetic operations, where each multiply-accumulate (MAC) is counted as two operations (one multiplication and one addition). The summation is taken over all *l* layers of the CSSNN. For each layer, *H*_*out*_ and *W*_*out*_ represent the spatial height and width of the output feature map, respectively; *C*_*in*_ and *C*_*out*_ denote the numbers of input and output channels; and *K*_*H*_ and *K*_*W*_ are the height and width of the convolutional kernel.

For FC layers, *H*_*out*_ = 1, *W*_*out*_ = 1, *K*_*H*_ = 1, and *K*_*W*_ = 1, reducing the expression to the standard dense layer operation count.

### 4.2 Network optimization procedure and result

In this experiment, we evaluate the performance of the CSSNN method under different CRs with different measurement matrix sparsity on three datasets. All the experiments are based on the SpikingJelly framework (Fang et al., 2023).

In this experiment, all input data are compressed using uniform CRs of 0.1, 0.05, 0.025, and 0.01. For each CR, the measurement matrix is further sparsified by controlling the number of ones (*Sample*) in each row, with corresponding sparsity levels of 0.1, 0.05, 0.025, and 0.01.

For all three datasets, the classifier is a three-layer fully connected SNN with a hidden layer size of 64. For all the neurons in the SNN, the MP threshold *v*_*th*_ is 1.0, the MP reset *v*_*reset*_ is 0, and the membrane leakage time constant τ is 2.0. The surrogate function used in STBP is the arctangent-based surrogate function, defined as ATan(α = 2.0).

The detailed structures of the CSSNN on three datasets are shown in Table 3.

**Table 3:** Network structure details.

Dataset	Baseline	CSSNN
MNIST	784-fc64-fc10	784-cs(784 × CRs)-fc64-fc10
N-MNIST	2,312-fc64-fc10	2,312-cs(2312 × CRs)-fc64-fc10
DVS Gesture	2 × 128 × 128-fc64-fc11	32,768-cs(32,768 × CRs)-fc64-fc11

fc, full connection; cs, compressed sensing.

For all the networks, adaptive moment estimation with weight decay (AdamW) is used as the optimizer, and the learning rate (LR) of each parameter group is decayed by a factor of 0.1 with an initial LR of 0.001 per epoch. All the networks are trained for a total of 64 epochs, with a batch size of 18. The total number of simulation timesteps is 100 for the MNIST dataset, 10 for the N-MNIST dataset, and 16 for the DVS Gesture dataset.

#### 4.2.1 Classification performance

**Figure 8:**
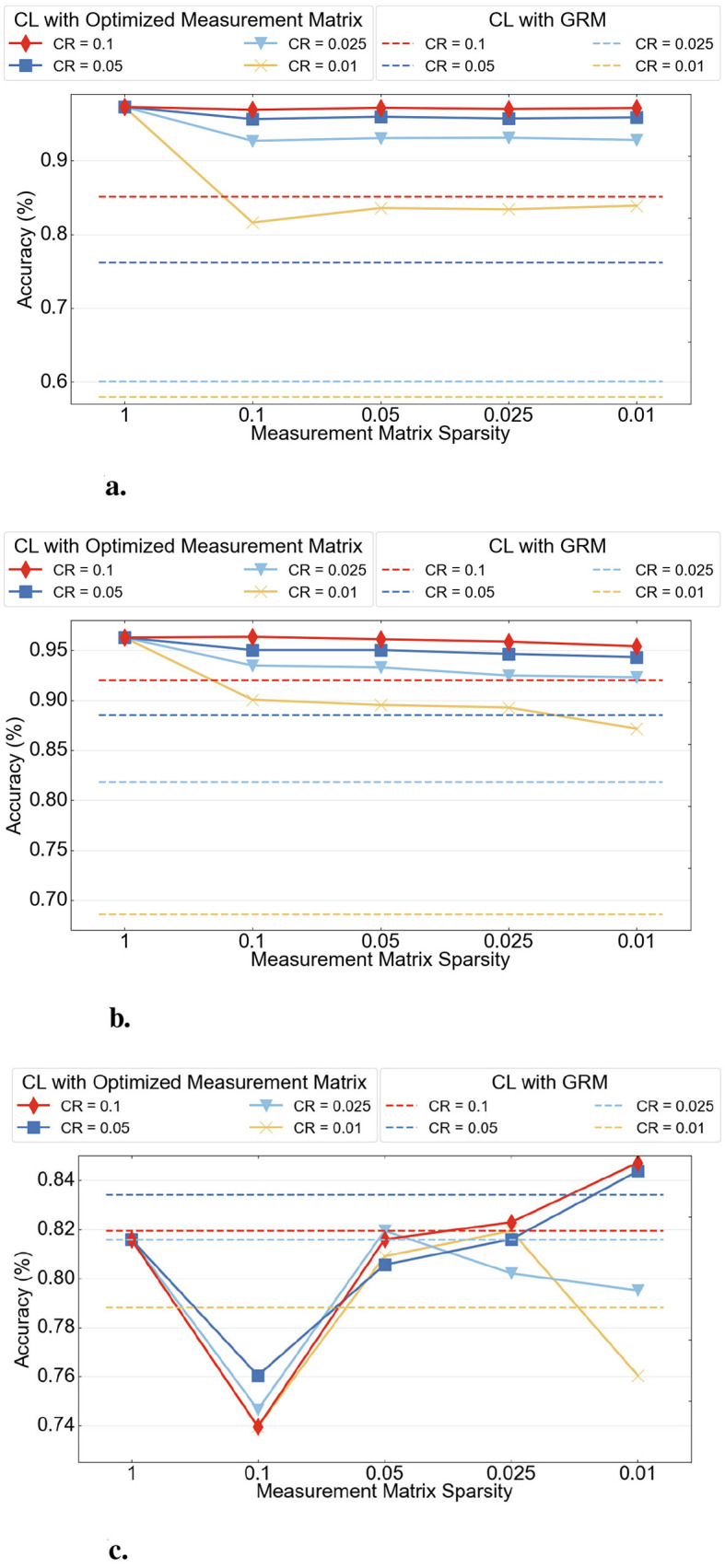
CSSNN classification performance on three datasets. **(a)** MNIST. **(b)** N-MNIST. **(c)** DVS Gesture.

The detailed classification performance using the CSSNN method on three datasets is shown in Figure 8.

In these figures, the first column shows the classification performance of the baseline model, which is a fully connected three-layer SNN, as reported in Table 3, while the dotted lines represent the performance of the CL method using a fixed GRM as the measurement matrix. The performance under different CRs is indicated by different colors.

In all experiments, the model does not require fine-grained hyperparameter tuning to achieve stable and consistent results. The key parameters, including the CR and the number of samples (*Sample*), have intuitive effects. CR controls the degree of information compression, while *Sample* determines the sampling density and sparsity of the measurement matrix. The measurement matrix sparsity is defined as $\frac{\text{Sample}}{n}$, where *n* is the original signal length. As illustrated in Figure 8, on the MNIST and N-MNIST datasets, classification performance decreases as the CR and measurement matrix sparsity decrease, indicating a smooth and predictable trend.

In contrast, for the DVS Gesture dataset, the accuracy increases as the measurement matrix sparsity decreases at CRs of 0.1 and 0.05. This is because DVS Gesture has higher input dimensionality and stronger sparsity, requiring finer CR and sparsity settings, where small parameter changes can lead to large variations in the Sample value. Meanwhile, the sparse data structure allows fewer sampling operations while preserving more informative events, requiring more accurate feature extraction.

Overall, across all three datasets, the CSSNN method consistently outperforms the CL method based on fixed GRM sampling matrices.

#### 4.2.2 Computational complexity

**Figure 9:**
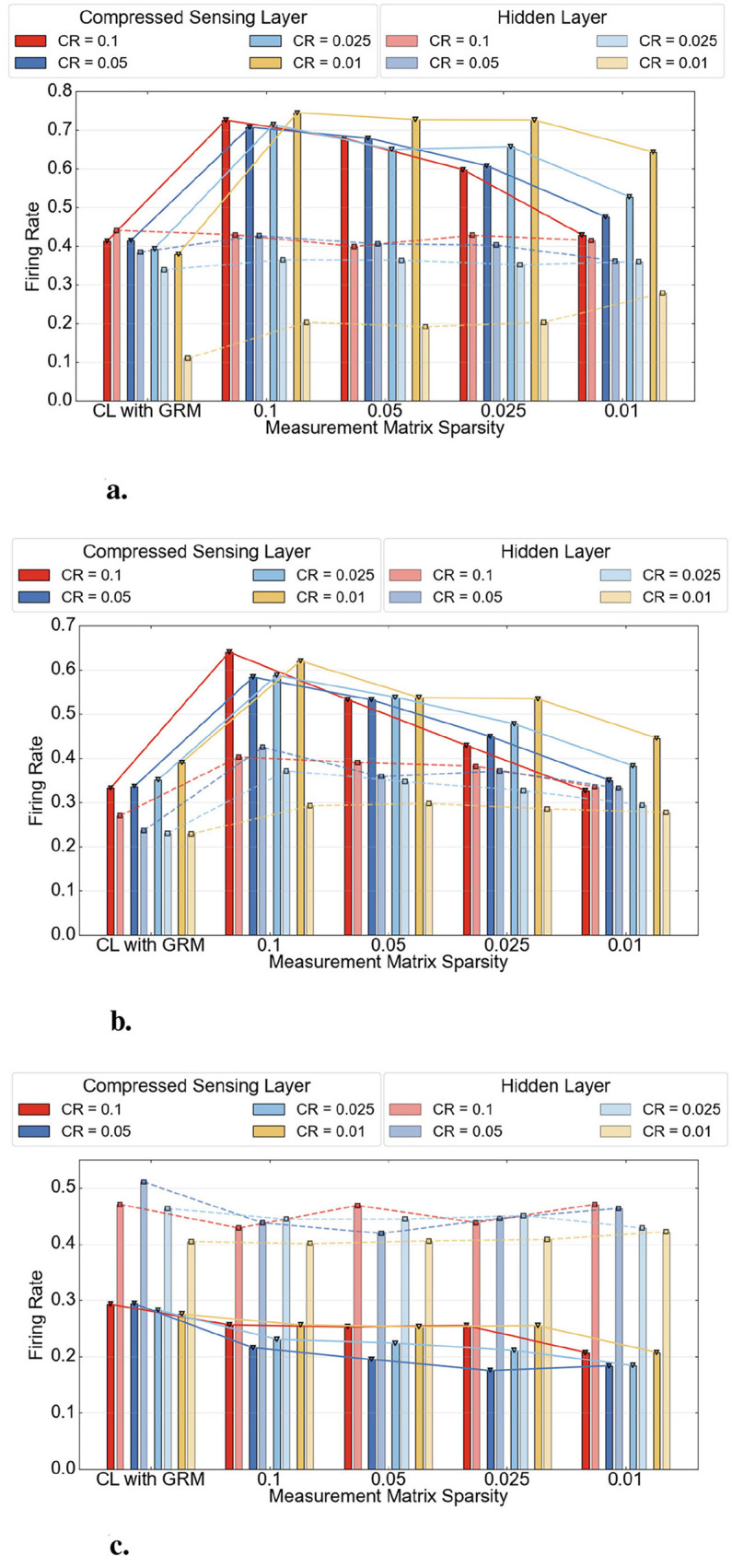
Firing rate performance of CSSNN on three datasets. **(a)** MNIST. **(b)** N-MNIST. **(c)** DVS Gesture.

To quantify the sparsity and event-driven characteristics of the proposed model, we report the detailed firing rates of both the compressed sensing layer and the hidden layers across three datasets, as shown in Figure 9.

In these figures, the first column shows the firing rate performance of the baseline CL method using a fixed GRM as the measurement matrix.

The firing rate behavior is influenced by both the structured sampling mechanism and the intrinsic sparsity of the input data. Under the same CR, reducing the sparsity of the measurement matrix decreases the number of sampled inputs per neuron, leading to lower accumulated input and reduced firing rates across all datasets.

However, different trends are observed with varying CRs. For MNIST and N-MNIST, the firing rate increases as the CR decreases under the same sparsity, due to the aggregation of informative signals in these relatively dense datasets. In contrast, for the inherently sparse and event-driven DVS Gesture dataset, reducing the CR lowers the probability of capturing informative events, resulting in decreased firing rates.

These results highlight the interplay between sampling structure and data characteristics in CSSNN.

**Figure 10:**
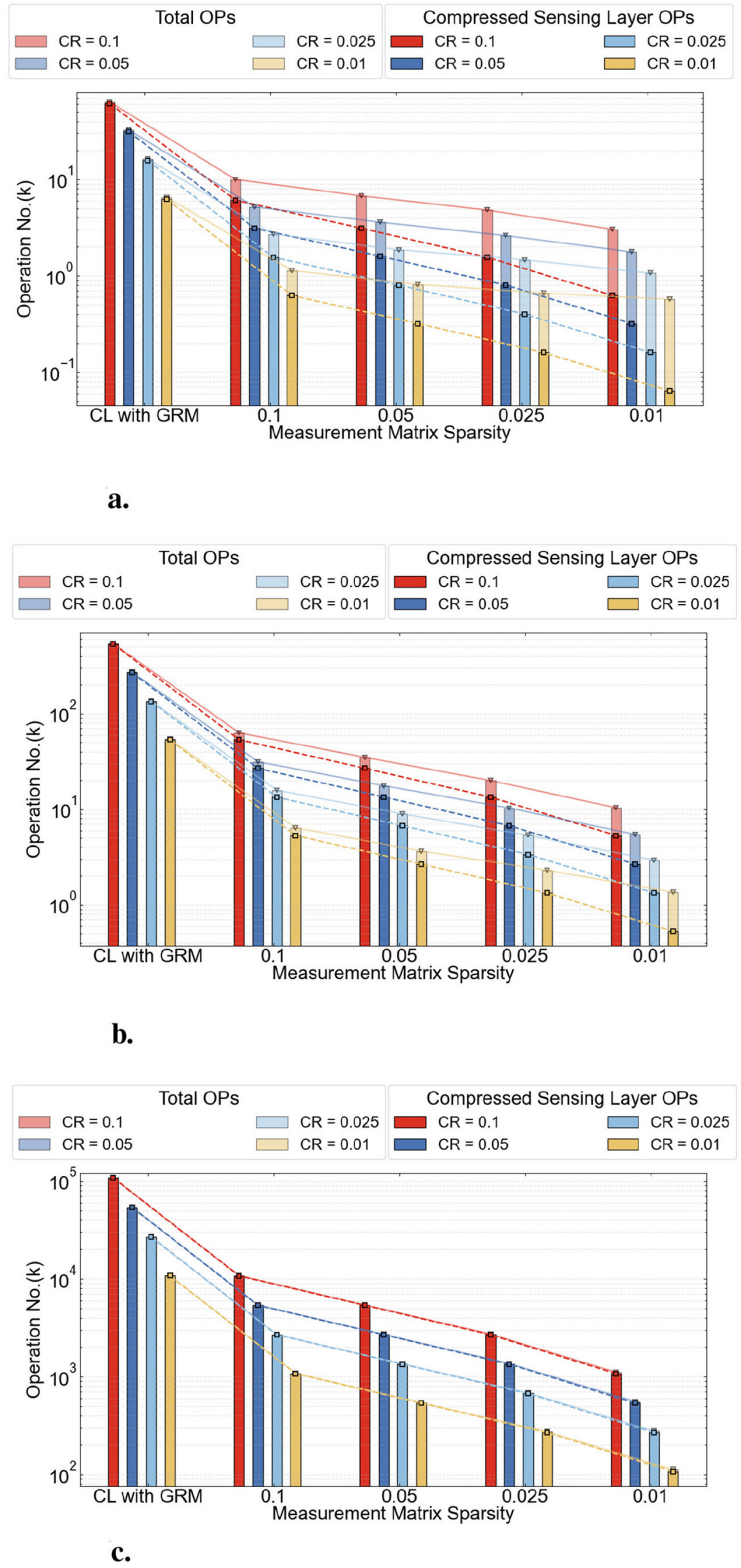
OPs of CSSNN on three datasets. **(a)** MNIST. **(b)** N-MNIST. **(c)** DVS Gesture.

The detailed OPs using the CSSNN method on three datasets are shown in Figure 10.

In these figures, the first column shows the OPs of the baseline network, namely the CL method using a fixed GRM as the measurement matrix. As shown in the figure, the majority of OPs are contributed by the CS layer. Therefore, as the CRs and the sparsity of the measurement matrix decrease, the overall number of OPs is significantly reduced. This trend is consistent across all three datasets.

Although in some cases shown in Figure 9 the baseline exhibits lower firing rates than CSSNN, the overall OPs of CSSNN remain substantially lower than those of the baseline. This reduction is primarily attributed to the sparsity of the measurement matrix, which significantly lowers the computational cost.

The proposed co-optimization does not introduce significant additional training overhead compared to baseline SNNs. The CS encoder is implemented as a lightweight linear operation and shares the same backpropagation procedure as the SNN. The primary additional cost comes from the sorting and selection operations used for binarizing the measurement matrix. However, the induced sparsity reduces computational complexity during the forward pass, leading to lower execution time and resource consumption than conventional SNNs. Therefore, the overall training complexity is comparable to, or even lower than, that of conventional SNN models.

For hardware deployment, considering that many SNN accelerators support up to 256 input channels, the CSSNN is configured as 784-cs256-fc64-fc10. For MNIST, the network parameters are quantized from 32-bit floating-point to 8-bit fixed-point using the BPSR training algorithm (Yan et al., 2022). The number of simulation timesteps is set to 10 to improve efficiency.

**Figure 11:**
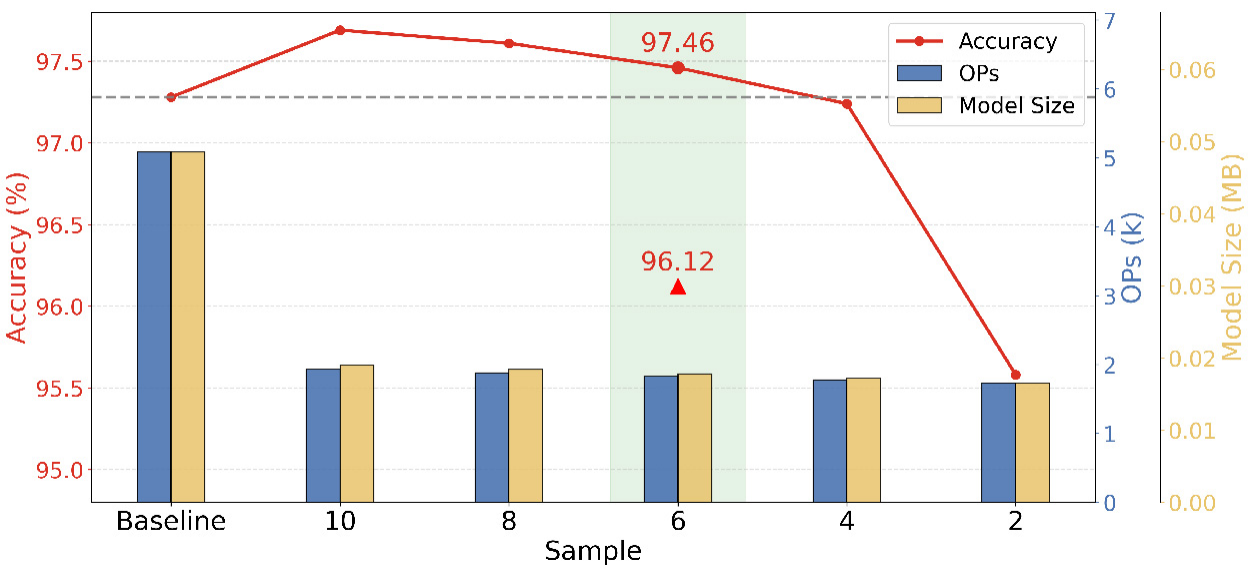
Performance trade-off under CS sparsity and quantization for hardware deployment on MNIST.

For the 784-cs256-fc64-fc10 configuration, we evaluate the impact of CS measurement sparsity by varying *Sample*. The trade-off among accuracy, OPs, and model size is shown in Figure 11. Based on this trade-off, *Sample* = 6 (green highlight) is selected as optimal, achieving 97.46% accuracy (32-bit baseline, dot marker) and 96.12% after 8-bit quantization (red triangle).

## 5 Hardware implementation and results

The proposed CSSNN processor for sparse signal classification is implemented using Vivado and verified on an FPGA platform. The ASIC design is synthesized and implemented by Synopsys Design Compiler and analyzed with PrimeTime.

### 5.1 FPGA prototyping

**Figure 12:**
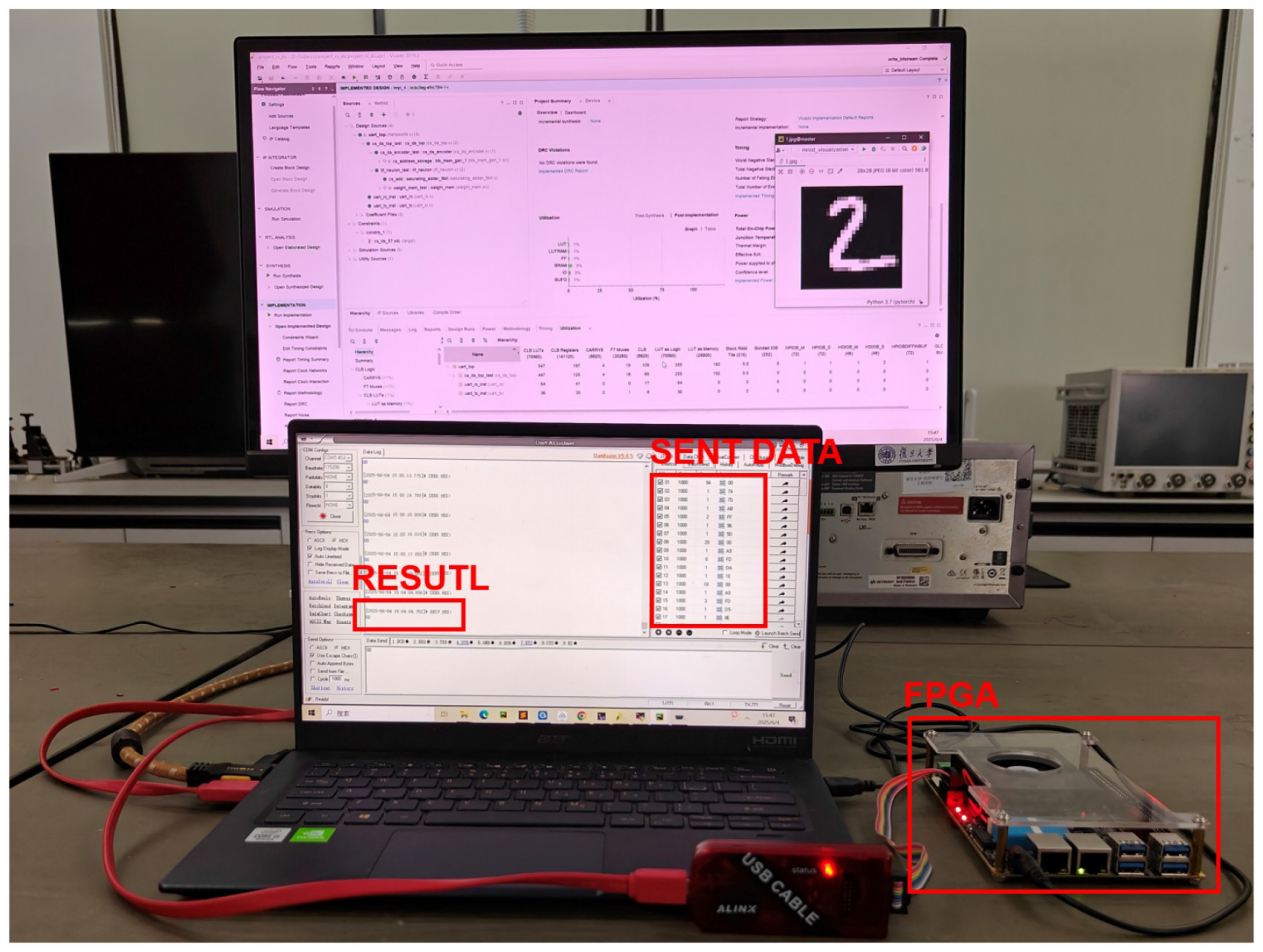
FPGA prototyping for the validation of the proposed CSSNN processor. A PC and an FPGA are employed to build the testing system. The PC generates image pixel data and sends it to the FPGA sequentially through the UART. The FPGA is implemented with the CSSNN processor to process the input data. The classification results are sent back to the PC through the UART.

As illustrated in Figure 12, this CSSNN processor is evaluated on the Xilinx Zynq UltraScale+ MPSoC XCZU3EG FPGA, with a PC acting as the host. The PC generates flattened MNIST pixel data and sends the data sequentially to the FPGA through the UART. The FPGA is implemented with the CSSNN processor, processing the input pixels and sending out the classification result. The result on the PC shows that the FPGA classifies the input image correctly. According to the trained measurement matrix, the last pixel sampled from the original data is number 770. Therefore, the FPGA sends out the classification result after 770 image pixels have been transmitted.

The resource utilization is listed in Table 4. The proposed CSSNN processor takes 547 look-up tables (LUTs), 192 LUT-based RAMs (LUTRAMs), and 197 flip-flops (FFs), which are all less than 1% of the available resources. A total of 5.5 block RAMs (BRAMs) out of 216 are utilized as the memory blocks, taking 2.55% of the total BRAMs resources. The memory consumption is calculated to be 0.193 MB. The comparison of the proposed CSSNN processor with other SNN classifiers implemented on the FPGA platform is demonstrated in Table 5. All the works presented use the fully connected SNN model.

**Table 4:** Resource utilization of FPGA.

Resource	Utilization	Available	Percentage
LUT	547	70,560	0.78%
LUTRAM	192	28,800	0.67%
FF	197	141,120	0.14%
BRAM	5.5	216	2.55%
I/O	5	252	1.98%
BUFG	1	196	0.51%

**Table 5:** Comparisons of proposed CSSNN FPGA implementation performance with related works.

Works	ISCAS'21	TCAS I'22	A-SSCC'22	TVLSI'22	TCAD'22	BioCAS'23	This work
	Sakellariou and Paliouras (2021)	Liu et al. (2022)	Fang et al. (2022)	Kuang et al. (2022)	Ye et al. (2022)	Besrour et al. (2023)	
Platform	ZCU104	Kintex-7	Alveo U250	KCU115	Kintex-7	ZCU102	XCZU3EG
Frequency (MHz)	100	200	75	140	200	-	200
Neuron number	4,096	16K	-	2,048	-	-	330
Synapse number (K)	524.29	16,000	-	512	-	-	17.02
SNN Model	784-fc300-fc10	784-fc1024-fc1024-fc10	784-fc1000-fc10	784-fc200-fc100-fc10	784-fc50-fc10	400-fc128-fc10	784-cs256-fc64-fc10
Weight precision	12-bit fixed	16-bit fixed	8-bit fixed	4-bit/8-bit fixed	16-bit fixed	8-bit fixed	8-bit fixed
Accuracy (%)	92.15	97.70	98.26	96.9	98.45	87.2	96.12
LUT	378,000 (75%)	46,371 (6.0%)	21,560	585,978	16,350	150,082 (54.76%)	547 (0.78%)
	691 ×	84 ×	39 ×	1071 ×	29 ×	274 ×	1
FF	216,670 (47%)	-	20,863	232,686	-	448,592 (81.84%)	197 (0.14%)
	1,099 ×	-	105 ×	1,181 ×	-	2,277 ×	1
B/URAM	0.55 (5%)+18.09 (67%)	150 (18.8%)	-	432	220	-	5.5 (2.55%)

fc, full connection; cs, compressed sensing.

All the other works exhibit LUT resource consumption more than 10 × higher and FF utilization more than 100 × higher than those of our work. In (Besrour et al. 2023), the input image is compressed from 784 to 400 to meet the hardware constraints. Consequently, the classification accuracy is reduced to 87.2%, which is 8.92% lower than that achieved by our method.

### 5.2 ASIC implementation

**Figure 13:**
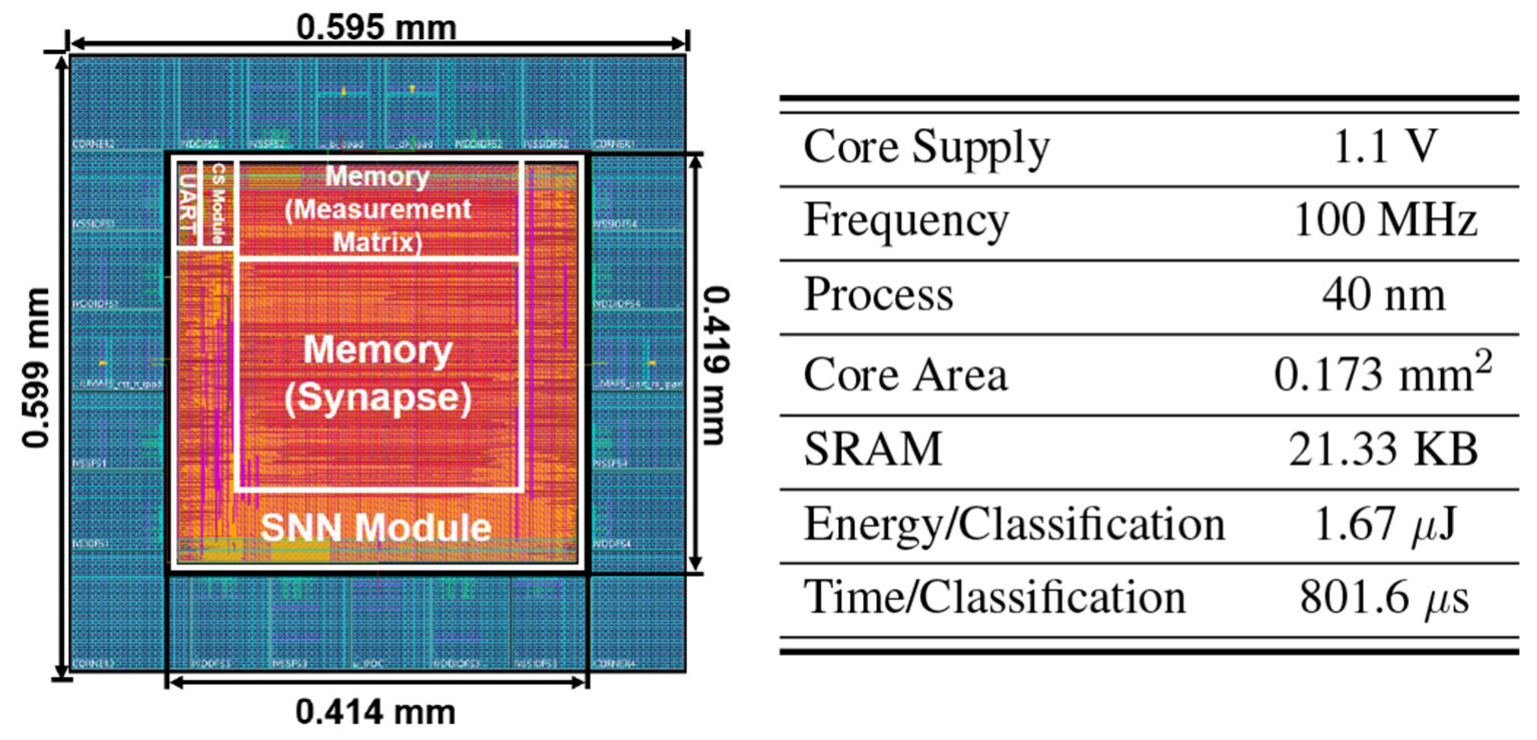
Layout and ASIC specification of the proposed CSSNN processor.

The physical design and the layout of the proposed CSSNN processor are accomplished by Cadence Innovus with a 40 nm CMOS process. The synthesized netlist is generated using Synopsys Design Compiler, and the post-layout power consumption is analyzed with Synopsys PrimeTime. As shown in Figure 13, the core processing area of the ASIC excluding I/O pads is 0.414 mm × 0.419mm, taking up 0.173 mm^2^. As shown in the layout results, the two SRAMs take up the most area. The SNN module takes a much larger area than the CS encoder module due to the neuron state memory block. The proposed CSSNN processor consumes 2.089 mW at a clock frequency of 100 MHz and a supply voltage of 1.1 V.

This reported post-layout ASIC results correspond to the complete processor core implementation. The design includes all core modules described in the architecture, namely the CS encoder module, SNN module, measurement-matrix SRAM, synapse SRAM, FSM/control logic, and UART interface. Accordingly, the reported area and power reflect the full on-core implementation of the proposed design. The I/O region, including I/O pads and associated overhead, is not included in the reported results. Since the design has not been fabricated as silicon, a complete system implementation would additionally require chip-level integration of peripheral circuitry and I/O interfaces.

The ASIC implementation is evaluated on MNIST as a representative benchmark due to practical hardware design constraints. The proposed CSSNN architecture is data-agnostic and depends primarily on the input dimensionality, CR, and measurement matrix sparsity. For N-MNIST and DVS Gesture, the CS encoder module maintains the same logic and hardware structure.

The primary difference lies in memory requirements, which dominate the chip area as demonstrated in Figure 13, and scales with CRs, measurement matrix sparsity, and input size as illustrated in Figure 4. Accordingly, the energy consumption follows the number of operations as shown in Figure 10, where the CS layer scales with input size, CRs, and sparsity, while the SNN layer scales with the firing rate.

The comparison of the proposed CSSNN processor design with other related works is presented in Table 6.

**Table 6:** Comparisons of proposed CSSNN hardware implementation performance with related works.

Works	TCAS II'21	TVLSI'24	APCCAS'24	This work
	Nambiar et al. (2021)	Li et al. (2024)	Wang et al. (2024)	
Process (nm)	40	40	28	40
Weight precision	8-bit fixed	5-bit fixed	8-bit fixed	8-bit fixed
Area (mm^2^)	4.8	16.3	8.66	0.173
Accuracy (%)	97.86	96.11	97.1	96.12
Supply voltage (V)	1.1	0.85	0.6	1.1
Frequency (MHz)	133	185	124	100
Power total (mW)	28.515	17.1	22	2.089

According to Table 6, our work has the smallest core area among all the works. Under a comparable supply voltage of 1.1 V and an operating frequency of 100 MHz, our work has the lowest power consumption. In Nambiar et al. (2021), the chip consists of 16 PE cores with a 128 × 128 crossbar array of 8-bit synaptic weights on each core. While implementing a four-layer (i.e., 784-fc128-fc128-fc10) DoReFa network (Zhou et al., 2016) to accomplish the MNIST classification task, at least 8 cores are required. Given a single core power consumption of 13.4μW/core/MHz, under 1.1 V and 133 MHz, the minimum power consumption is calculated as 14.257 mW. Although (Wang et al. 2024) achieves a 0.98% higher classification accuracy, it incurs approximately 10 × higher power consumption and a core area that is about 50 × larger.

## 6 Discussion

There is a growing demand for lightweight SNNs suitable for deployment on resource-limited edge devices. The results of this work indicate that combining CS with SNNs is an effective strategy to address this challenge. By jointly optimizing the measurement matrix and the SNN classifier in an end-to-end fashion, the proposed CSSNN enables task-adaptive sensing that directly aligns with the classification objective. Unlike conventional learned encoders or BNN-style front-ends, the binary {0, 1} measurement matrix in CSSNN models a structured sampling process, where each measurement selects a fixed number of input elements under a column-wise constraint, forming a fixed sampling cost. This design preserves a non-negative accumulation property, thereby improving hardware efficiency, while enabling effective compression with minimal accuracy loss. Across the MNIST, N-MNIST, and DVS Gesture datasets, under consistent CRs of 0.1, 0.05, 0.025, and 0.01, the proposed CSSNN demonstrates a significant reduction in the total number of network OPs, achieving at least an 80% decrease compared with compressed learning approaches based on fixed GRM sampling matrices. A dedicated neuromorphic processor is designed to support the CSSNN process with on-chip spike encoding via an index-matching CS encoder module and an SNN module. Evaluated on the XCZU3EG FPGA platform, the LUTs, LUTRAMs, FFs, and BUFG utilizations are below 1%. The CSSNN processor design is synthesized and implemented using the 40 nm CMOS process. The power consumption is 2.089 mW under a 1.1-V voltage and 100 MHz frequency, occupying a core area of 0.173 mm^2^.

## Data Availability

The original contributions presented in the study are included in the article/supplementary material, further inquiries can be directed to the corresponding authors.
